# Profiles of palliative day care programs in Canada and the United Kingdom: A meta-synthesis

**DOI:** 10.1177/26323524251383031

**Published:** 2025-10-14

**Authors:** Gabrielle Fortin, Gabrielle Leblanc-Huard, George Kernohan, Felicity Hasson

**Affiliations:** 1 School of Social Work and Criminology, Laval University, Québec City, QC, Canada; 2 Institute of Nursing and Health Research, School of Nursing and Paramedic Sciences, Ulster University, Belfast, UK

**Keywords:** palliative day care programs, palliative care, meta-synthesis, cross-country analysis

## Abstract

**Background:**

The first palliative day care program (PDCP) marks its 50th anniversary.

**Aim:**

This study examined the distinctive features of PDCPs that have endured, as well as the changes they have undergone in the United Kingdom and Canada, to identify avenues for the development of these programs.

**Methods:**

Using primary data from two qualitative studies, conducted in the United Kingdom and Canada, a thematic meta-synthesis was carried out using the expansive secondary analysis approach to identify similarities and distinctions between the PDCPs identified in the two original studies.

**Results:**

The results were drawn from group and individual interviews with 19 participants in Canada, including 13 professionals and 6 managers across 6 PDCPs, and 35 participants in the United Kingdom, including 16 professionals and 18 managers from 3 PDCPs. The results indicate that the administrative structure of the PDCPs, the adoption of a palliative care philosophy, and the multidisciplinary nature of the professional and volunteer teams are the components of the programs that have endured. However, patient characteristics, care models, and institutionalization are constantly evolving.

**Conclusions:**

As PDCPs continuously innovate to adapt to the needs of their patients, the evolution of their components is desirable. However, pressure to demonstrate the relevance of their services to justify financial resources could, while ensuring their sustainability, deprive them of the values and practices that are their most valuable asset and purpose: supporting people living with advanced diseases with palliative care while still remaining in their own homes.

## Plain language summary

Palliative day care programs in Canada and the United Kingdom

Palliative Day Care Programs (PDCPs) offer people with advanced illnesses a place to receive medical, social, and emotional support while continuing to live at home. Fifty years after the first PDCP opened, this study examined which features of these programs have remained the same and which have changed, through an international comparison of four PDCPs in the United Kingdom and five in Canada. Using secondary analysis of 54 interviews with managers and professionals, we found that core elements—such as administrative structure, palliative care philosophy, and multidisciplinary teams—have endured over time. However, the populations served, models of care, and degree of institutionalization continue to evolve. While adapting to patients’ changing needs is essential, increasing pressure to demonstrate value for funding could unintentionally undermine the social and community values that make PDCPs unique, and that help people live meaningfully at home despite advanced illness.

## Introduction

Although there is currently no formal definition underpinning palliative day care programs (PDCPs), it is recognized that they are places where people with advanced illnesses can visit during the day to receive a range of psychosocial, physical, and spiritual care to improve their quality of life.^1^ The first PDCP was founded in 1975 in the United Kingdom by St. Luke’s Hospice in Sheffield.^2^ This program offered a range of support services and physical care to people with cancer and chronic illnesses.^3^ Since its inception, the PDCP model has grown considerably in the United Kingdom: by 2008, there were over 280 programs, adopting a predominantly medical, social, or mixed model of care.^4,5^

The history of PDCPs in Canada is closely linked to that of the United Kingdom. The Bonenfant-Dionne Centre was the first PDCP to be established in 2000. It was largely inspired by St. Christopher’s Hospice, a UK-London-based PDCP founded in 1990, which promoted a psychosocial approach to palliative care.^6,7^ The Bonenfant-Dionne Centre served as a model for the five other PDCPs (Number of known programs at the time of the Quebec post-pandemic study in 2022) that have since opened their doors in Canada, all located in the province of Quebec. There is, therefore, a historical and organizational proximity between UK and Canadian PDCPs.

Early research indicates that the scope, scale, and services provided by PDCPs in the United Kingdom vary.^8–11^ To date, the description of PDCPs remains sparse and outdated, with no international comparisons existing in the evidence base. The aim of this article is, therefore, to take a cross-country look at the evolution of PDCPs in the two countries, drawing on qualitative data from two research projects, to identify avenues for reflection on the future of PDCPs. The evolution of PDCPS, frameworks, current provision, and challenges and facilitators within each healthcare system will be described and compared. This paper presents a building block for enhancing understanding for possible future research.

## Methodology

Upon reading an article by Hasson et al.^1^ on the realities of PDCPs in the United Kingdom, a team of Canadian researchers conducting a similar study on Canadian PDCPs^12^ became curious about the evolution of Anglo-Saxon PDCPs. The Canadian team contacted the UK research team and proposed a collaboration on this article. The results of this article are drawn from a meta-synthesis of the data included in the two research projects they carried out. The reporting of this study conforms to the ENTREQ statement (Supplemental Material).^13^

### Qualitative research conducted in Canada

A descriptive, qualitative study, conducted between November 2022 and June 2023, examined the professional practices and services of PDCPs to better understand their impact on service users.^12^ Two days of workshops were conducted with PDCP coordinators. Subsequently, qualitative semi-structured interviews were conducted with professionals from five PDCPs to document the models and practices they recommend and to identify the perceived benefits and challenges in their daily practice.

### Qualitative research conducted in the United Kingdom

As part of larger-scale evaluative research, a qualitative, exploratory study was conducted from 2016 to 2019 to understand, from the perspective of management teams and professionals, how the PDCPs contributed to the care delivered to patients.^1^ Six exploratory focus groups were conducted in three PDCPs run by the Marie Curie Foundation.

### Secondary data analysis

To pool the data and examine the results of the two studies,^14–16^ a secondary analysis of the qualitative data was undertaken. An analytic expansion approach^17^ was chosen to question the existing qualitative data from the two research projects to answer two new research questions: (1) are there common foundations and values between PDCPs across continents? and (2) how can the evolution of UK and Canadian PDCPs help identify future avenues for the recognition and development of PDCPs?

To conduct the secondary analysis of the qualitative data, the two research teams met by videoconference in June, July, and August 2023. The UK research team’s focus group transcripts were shared with the Canadian research team, who carried out a thematic analysis following the method proposed by Paillé and Muchielli.^18^ Each verbatim was coded by paragraph to search for concepts. Recurring themes, categories, and subcategories were identified and integrated into a thematic tree. The categories of analysis were defined according to a mixed model, in which the first portion of the categories was drawn from the original studies, and the second portion emerged during analysis to integrate themes that would identify commonalities and differences between PDCPs in Canada and the United Kingdom.^19^

## Results

This section begins by describing the participants of the two research projects. The results are then presented according to two main themes: (1) the foundations of PDCPs and (2) the evolution of PDCPs.

### Participant profile

In Canada, 2 workshops were held with 6 PDCP coordinators, followed by 13 interviews with professionals (Table 1). In the United Kingdom, a total of 35 people, including 18 managers and 16 professionals, were interviewed (Table 2).

**Table 1.** Profile of Canadian participants.

Site	Number of participants	Designations
1	4	Manager, Art Therapist, Nurse, and Social Worker
2	3	Manager, Nurse, and Yoga Therapist
3	4	Manager, Acupuncturist, Social Worker, and Music Therapist
4	4	Manager, Social Worker, Physiotherapist, and Occupational Therapist
5	4	Manager, Nurse, Art Therapist, and Massage Therapist
6	1	Manager

**Table 2.** Profile of United Kingdom participants.

Site	Focus group number	Number of participants	Designations
1	1	3	Lead Nurse, Day-Services Manager, and Hospice Manager
	2	7	Healthcare Assistant, Staff Nurse (*n* = 2), Consultant, Physiotherapist, Occupational Therapist, and Volunteer Coordinator
2	3	10	Principal Social Worker, Community Services Lead Nurse, Community Services Manager, Hospice Manager, Clinical Specialist Services Manager, Palliative Care Consultant, Administrative Advisor, Allied Health Professional Manager, Manager, Lead Nurse, IPU and Day-Services Manager
	4	9	Healthcare Assistant, Staff Nurse, Physiotherapist, Volunteer, Community Nurse Specialist, Social Worker, Occupational Therapist, Volunteer Complementary Therapist, and Community Nurse Specialist
3	5	3	Staff Nurse (*n* = 2) and Occupational Therapist
	6	3	Day-Services Sister, Hospice Manager, Palliative Care Consultant

### The foundations of PDCPs: An unaltered core

Since the creation of the first PDCP nearly 50 years ago, many components of this model of care have evolved. However, certain characteristics have persisted over time in both Canada and the United Kingdom. The administrative model, the philosophy of palliative care, and the composition of professional teams form a solid foundation at the heart of the unique contribution that PDCPs make to the lives of people with advanced illness and their families.

#### Administrative structure of programs

In both Canada and the United Kingdom, the vast majority of the PDCPs studied are managed by nonprofit organizations, with approximately two-thirds of their funding coming from donations or other private sources and one-third from public sources. Also, the vast majority of PDCPs included in the study were attached to inpatient hospices, often sharing common facilities. They serve people with serious illnesses with a prognosis of more than 2 months, thus serving a broader clientele than the hospices to which they are affiliated. By offering services that complement public services and hospices, PDCPs play an important role in the continuum of integrated palliative care, supporting service users who continue to live at home in their communities before the end-of-life period.

#### The philosophy of palliative care

Another lasting characteristic is the organizational culture of the PDCPs, which is based on the philosophy of palliative care. Despite some differences, all PDCPs adopt the values and ethics of this philosophy and view their mission as improving the quality of life and well-being of people with serious and incurable illnesses. As the testimonial from this social worker shows, this quality-of-life approach often enables people to remain in their own homes for longer if they wish:

It’s improving the quality of life for people and often enabling them to remain at home longer than they maybe would have been able to if they didn’t have support either from day services or the community team. (Social worker, PDCP 2—United Kingdom)

To respond to the needs of their service users and support them, all PDCPs claimed to deploy person-centered care in a holistic approach aimed at meeting all their biopsychosocial and spiritual needs. PDCP professionals reported a need to be highly flexible, adapting their services to the needs of everyone.

The palliative care philosophy adopted by the PDCPs placed great emphasis on the social dimension of health. A strong emphasis is placed on peer support to reduce isolation and enable patients to share their experiences:

I think peer support for patients, that’s one big strength of day services. Speaking to somebody who is in the same boat as they are or maybe having the same illness sometimes helps them to cope a wee bit better. (Physiotherapist, CJSP 2—United Kingdom)

Another distinctive feature of the palliative care philosophy reported by the PDCP staff is that they are rooted in their communities. The programs are constantly innovating to adapt their services to their geographic and demographic realities. In some PDCPs, satellite locations have been opened to bring services closer to people’s homes and thus improve accessibility of the programs. Other PDCPs adapt their services to the characteristics of their service users by establishing, for example, days dedicated to young adults, as this social worker puts it:

Wednesday is young adults’ day because there’s a support group for young adults, so particularly for young adults if they feel drawn to be with other young people. (Social worker, PDCP 1—Canada)

The philosophy of palliative care means that many programs welcome not only the service user but also their loved ones. In most of the PDCPs, individual or group services were offered to users’ family members to provide support, teaching, and respite. These services are one of the distinctive features of PDCPs compared with other palliative care services, which generally focus on the user alone:

That’s one of the biggest impacts I’ve found for community patients. Day services have been able to offer families and carers complementary therapy because it’s huge to be able to say if the patient doesn’t want to or doesn’t need to come to day hospice or day services, but the partner or daughter can come. (Community nurse specialist, PDCP 2—United Kingdom)

This nurse’s testimony shows the importance of providing services to the loved ones who attend the programs so that they can restore a state of well-being that will enable them to be more present for their loved ones. Also, by offering services to users and their loved ones, the PDCPs create a form of community life that can facilitate the transition of loved ones to bereavement services after the user’s death, as this nurse testifies:

A gentleman who had suicidal thoughts, his wife died nine months ago. But all the same, we opened the door. I asked him why he had come here. He said, “I just remembered that you said I’d be here if anything happened.” But it helped me make the connection with the bereavement follow-up afterwards. (Nurse, PDCP 5—Canada)

PDCPs are, therefore, a shared space where the loved one and the ill person can walk together, and important places of transition and reassurance for those who remain after the death.

#### The composition of professional teams

Since the creation of the first PDCP, the multidisciplinary nature of the professional teams has persisted over time. PCPDs in Canada and the United Kingdom hire a wide variety of professionals from diverse disciplines, such as nursing, social work, occupational therapy, and physiotherapy. Alternative therapies, such as art therapy, music therapy, aromatherapy, and yoga therapy, also play an important role. Moreover, volunteers play an important role in providing general and specialist support to patients:

[. . .] I bring in volunteers who also play music. [. . .] We have a [patient] whose sister is a music teacher at school, and she comes to give us workshops from time to time. She brings her percussion instruments and we sit in a circle here and she makes us guess songs and then we play the instruments. It’s very simple, but it's enjoyable. (Music therapist, PDCP 3—Canada)

We are very lucky, and that would be one of the good things about day services, you know, we have the volunteer complementary therapists, we have the volunteer drivers for patients who can’t bring themselves, we have the volunteers who give out the tea and coffee and the food. So, we’re very lucky with the support that we get from the volunteers, and I suppose what we do we couldn’t do without the volunteers across all five days. (Staff nurse, PDCP 1—United Kingdom)

The experience of these two professionals underscores the importance of volunteer involvement. The freedom of intervention and absence of hierarchy that often characterize the professional dynamics of PDCPs facilitate the inclusion of volunteers. It is, therefore, possible to conclude that, after 50 years of existence, the PDCPs have retained several key components. In addition to anchoring their services in the community and deploying person-centered care, the presence of informality and the absence of hierarchy between the various practitioners foster a caring climate where people with advanced illness and their loved ones can find well-being.

### The evolution of PDCPs

Several aspects of PDCPs have changed significantly over the years. These changes, which are more pronounced in the United Kingdom than in Canada, relate to the characteristics of the programs’ clientele, the medicalization of services, and the level of institutionalization.

#### Clientele characteristics

Since their beginnings, the majority of PDCPs have served people with advanced cancer. However, for several years now, PDCPs have been opening their doors to people with other diseases with a poor prognosis. PDCPs that took part in the UK study all cared for people on a palliative trajectory with diagnoses other than cancer, as shown by the testimony of this occupational therapist:

. . .now we have done the re-branding of the service into any, sort of, end-of-life. It’s not just cancer care. So, we’ve opened the gates, I suppose, to many conditions, so there’s a lot more, I suppose, variety of symptoms and needs. (Occupational therapist, PDCP 1—United Kingdom)

In Canada, PDCPs are slowly opening to other client groups. At the time of the Canadian study, two of six existing programs were accepting users with advanced illnesses other than cancer.

Another evolution in the PDCP clientele is the growing needs of users. Professionals in Canada and the United Kingdom have noted that, over the years, their users have come to their services with increasing levels of illness:

Patients are becoming more ill and more complex and with more needs. [. . .] I’m not in day hospice since a long long time, but the difference from when I started, maybe a year and a half ago to now, I can see a huge change already in the complexity of the patients, the needs of the patients, the follow-up and liaison that is required [. . .] What I mean is, it’s definitely coming away from the “tea and scones.” (Staff nurse, PDCP 1—United Kingdom)

Although this nurse points out that she has not been working in PDCPs for long, she has been able to observe an increased complexity of illness among the people admitted to the programs. This reality is a challenge for PDCPs because as people attend their services for shorter periods before being transferred to end-of-life care, program managers must constantly be on the lookout for new service users and make many admissions so that their services are used to their full capacity, as this assistant puts it:

[. . .] one of the challenges of day services is actually trying to keep a full complement of patients coming [. . .] people come in and they leave for whatever reason [. . .] maybe a few people have died, and our numbers are down again and it’s this constant . . . it’s almost as if the service isn’t fully utilized. (Health care assistant, PDCP 2—United Kingdom)

This worsening of health problems among service users has contributed to another development at PDCPs: the medicalization of their services.

#### Medicalization of services

In recent years, several programs have established or are planning to open medical clinics to treat specific symptoms such as pain or shortness of breath. Symptom assessment is becoming an increasingly important part of the services offered by these programs. It is now common to have doctors assigned to the PDCPs, and nurses are taking on a predominant role, with many holding management positions. This progressive medicalization is distancing the PDCPs from their original social approach, as this doctor notes:

I’m here [since] nineteen years. But it was more a “tea and buns” kind of thing definitely to begin with. A big social dynamic and very little in the way of sort of clinical services, so there is a much much stronger clinical bent to it now. (Doctor, PDCP 1—United Kingdom)

As a result of this change, most PDCP managers in the United Kingdom recognize that they operate on a hybrid model combining both psychosocial and medical services, as this manager testifies:

Is it a social model or is it a medical model, I would like to think that we are 50/50, but more gearing towards the specialist model of symptom management, rehab, psycho-social support, education, promotion of health, all of that. (Day hospice manager, PDCP 1—United Kingdom)

This hybridization and medical specialization, while important for holistically meeting patients’ needs, is resource-intensive. As advanced medical services are often costly, an appointment-based system is frequently favored to maximize efficiency. However, this method of operation reduces the flexibility of services and leaves less room for informality, spontaneous exchanges, and the development of community spirit. Many participants in the UK study found the hybridization of medical and social models necessary but feared that services would be compromised due to a lack of resources and that the palliative care philosophy at the heart of the PDCP model would gradually disappear. The testimony of this nurse reveals the unease she feels about the fact that she no longer has time to devote to informal exchanges because of the growing demand for clinical care:

I’m starting to feel nearly a guilt. . .on a Wednesday especially it is very clinical and very very busy and, you know, I’m finding I have less and less time to sit. Some of the patients who are maybe less symptomatic or need, you know, less nursing attention, I barely get to say hello to them because I am just so taken up with patients who, you know, need their tasks doing or need assessed or obviously you’re liaising with the team. So, it’s definitely getting busier and much more clinical. (Staff nurse, PDCP 1—United Kingdom)

Others also mentioned feeling obligated to adopt an increasingly medical model due to the growing pressure from commissioners toward this type of care, as mentioned by this manager when talking about the model of care adopted by the PDCP where he works:

And our commissioners are more or less dictating too about how they want to see day hospice model going. And, there is money coming up and available to invest in day services, but in a certain way. [. . .] So, that’s one of the things that impedes us as well, is the commissioners impeding the service that we want to deliver by saying “we want it this way” But we’re saying “you can’t just have this medicalized model” in day service. You have to, especially in palliative care, it’s neither black nor white, there’s always grey. And you can’t treat two lung cancer patients the same or two breast cancer patients the same, because they’ve got all different needs and different social and economic backgrounds and dependencies. [. . .] with the commissioner’s input and with pressure from funding, you end up “Oh yes, we can do that, and we can do that, and we can do that,” and before you know it, you are so far away from where you started off. (Hospice manager, PDCP 1—United Kingdom)

It is apparent that medicalization, while desired by professional teams to meet the full range of user needs, also constitutes a threat to the unique PDCP model rooted in a social and community perspective.

In addition, the medicalization of PDCPs has introduced a logic of rehabilitation. Indeed, medical services at UK PDCPs are often intended to help users recover rather than to help them maintain a good quality of life on a long-term basis. Because of this, participants in the UK study report that their PDCPs are implementing discharge policies. When the user’s physical symptoms improve, the PDCPs terminate services, as this nurse testifies:

If you get it right, it certainly does work effectively, and we’ve certainly had people who’ve gone out there and got on with the rest of their life for a bit and that’s been very advantageous. We do try and sell discharge in a very positive manner; it’s not always accepted that well by the patient. If people are well and are stable then we do discharge them, they don’t take it very well always because they come and have a nice day out. (Staff nurse, PDCP 3—United Kingdom)

In Canadian programs, there is still no discharge policy, which is partly due to the advanced stage of many patients’ illnesses. However, medicalization and the rehabilitation logic are not the only developments threatening the social aspect of the programs. Participants also mentioned institutional constraints.

#### Institutionalization and funding of PDCPs

Several PDCP managers have reported feeling pressurized to institutionalize their programs to secure more stable funding from public authorities and ensure their sustainability. To achieve this, PDCP managers need to find ways to prove their effectiveness through an accountability system that measures the benefits of their services. However, the social and spiritual aspects of PDCPs and their impact, such as reducing isolation or alleviating existential anguish in the face of death, are difficult to quantify, as these participants testify:

I think what’s really interesting is that when you ask the question of what we have and what we do, when you talk about these sorts of things, and you can make them real with stories it’s pretty evident to other people what can be achieved. But trying to make that understandable for the people who actually fund and commission healthcare is actually very difficult because, we’re talking about seeing more people and seeing them earlier and providing this benefit and getting involved. . .. (Consultant, PDCP 2—United Kingdom)

We’re thinking, the whole gang, about how to make our stats a little richer so that we can precisely state all those grey areas where we’re helping patients, but I’m not pragmatically putting needles into their bodies, but I am working on two hours of sound editing to make their legacy as beautiful as possible, so that when I put it to the patient’s ears, they cry so much, they find it beautiful, it’s real work. And it has a real therapeutic effect, so we’re starting to work on how we can record it so that it’s visible. (Acupuncturist, PDCP 3—Canada)

Furthermore, the development of a standardized reporting tool for all PDCPs would jeopardize the community aspect of the programs since this tool would require standardization of services, making it more difficult to adapt them to users’ needs. The challenge for PDCPs in both Canada and the United Kingdom will be to prove the relevance of their model to public authorities while maintaining their autonomy so that they can continue to offer comprehensive care consistent with the philosophy of palliative care.

## Discussion

### Main findings

This article critically examines the evolution of PDCPs in Canada and the United Kingdom over the past 50 years to assess experiences and shape the future of these programs. Across both countries, findings indicate that PDCPs play a key role in the maintenance of the quality of life for patients and their families. Over the years, the administrative structure, the philosophy of palliative care, and the composition of the professional teams, despite some minor changes, have remained essential components of the PDCPs. However, the results also point to a weakening of these elements, notably due to the increasing burden of users’ illnesses, increased medicalization, and institutionalization of the programs.

### Implications for practice

With an aging population and a rising prevalence of serious illnesses, the demand for palliative care is expected to double over the next 40 years.^20^ PDCPs are, and will increasingly become, key actors in the continuum of integrated palliative care, offering complementary services to inpatient units, home care, and hospices.^6,21,22^ In this context, expanding this model of care is a pressing need. However, to date, no study has been able to demonstrate the effectiveness of PCDCs^21,23–25^ despite numerous qualitative studies reporting positive social and spiritual outcomes for patients and their families.^2,25,26^ As a result, public authorities remain hesitant to support PCDCs and provide them with minimal funding.^21^ Consequently, these centers rely heavily on private donations and volunteer services to sustain their activities.^6,8,24,27,28^ This financial instability—exacerbated by the decline in volunteer work since the COVID-19 pandemic^28^—restricts their capacity to hire maintain quality of care, hire additional professionals, expand services, and invest in new equipment or infrastructure. It is thus more difficult for PDCPs to innovate continuously and keep patient needs at the heart of their services. Developing accountability tools that can capture the social and spiritual added value of PDCPs—such as those explored in Bradley’s research,^29^ on the feasibility and acceptability of Patient-Reported Outcome Measures—should therefore be a priority for future studies.

Moreover, PDCPs provide either a medical, social, or mixed model of care, thus enabling the holistic needs of those living with and grieving to be met in the community. Demand for such services exceeds supply, with the need to expand this to other populations evident. The move toward a more medical model of care was recognized across locations. While this is deemed to be necessary to answer to increasing patients’ health needs, it may undermine the social element which is at the core of palliative care day programs and most valued by patients and their families.^9,25,26,30^ A qualitative study realized by Bradley et al.^31^ highlights the fundamental importance of the social dimension in PDCPs. The social support patients receive through interactions with other patients and staff fosters improved health by restoring their motivation and capacity to engage in daily activities. It also provides a safe and understanding environment in which they can openly discuss, plan, and define their end-of-life wishes without stigma or taboo.

The discharge policies implemented in the United Kingdom may signal a shift away from the social and spiritual dimensions that lie at the heart of the palliative care ethos. PDCPs’ holistic approach, rooted in person- and family-centered care, seeks to support individuals in living the remainder of their lives as meaningfully as possible.^32,33^ Because addressing psychological, social, and spiritual needs often requires ongoing support and accompaniment until the transition to end-of-life care, the discontinuation of services risks undermining the core values that guide PDCPs.

A mixed model of care, therefore, seems to be an important avenue for PDCPs to ensure a balance on the ethos of palliative care is assured and that over-medicalization may be avoided. However, it will be necessary to ensure that the medical care provided to patients is enhanced to meet their needs while maintaining an environment conducive to social interaction and person-centered care to meet all the biopsychosocial and spiritual needs of patients and their loved ones.^31,34^

### Limitations

It is important to identify the limitations of this article. Firstly, this is a pooling of two qualitative studies. While the Canadian study included all PDCPs in the country, the UK study selected only three sites across England, Scotland, and Northern Ireland, whereas there were over 250 (Unfortunately, more recent estimates of the number of PDCPs, particularly post-COVID, are not available yet) across the United Kingdom in late 1990s and early 2000s.^4,5^ There is no post-pandemic estimate of the number of PDCPs in the United Kingdom or indeed in Canada outside of Quebec. The results presented in this article, therefore, provide food for thought for the future of PDCPs but cannot be generalized. Secondly, the United Kingdom study was conducted from 2016 to 2019 and does not cover the pandemic period, whereas the Canadian study was conducted in 2022 and 2023. At the time of the interviews, patient attendance and operating procedures were still adapting to the upheavals of recent years. For this reason, the comparison of data from the two studies and the integration of themes related to COVID-19 were limited. Finally, while both studies were gathered the views of staff and managers, they did not seek the perspectives of service users and their families. Documenting these perspectives would be highly relevant for a future study.

## Conclusion

Based on the results of this cross-country meta-analysis of PDCPs’ evolution in Canada and the United Kingdom, we can conclude that, over the years, PDCPs in both countries have retained several essential components of their organizational model, such as multidisciplinarity, person-centered care, and adherence to the values of the palliative care philosophy. However, with the medicalization of programs, it is becoming increasingly difficult for them to find the resources to continue to offer quality services that meet the biopsychosocial and spiritual needs of service users and to continually innovate to adapt to the realities of their local communities. Given the projected rise of cancer and other chronic, incurable, and severe illnesses worldwide,^21,35–37^ we believe that PDCPs, with their unique and innovative community-based and patient-centered care, will be essential in improving the quality of life of patients and their loved ones and in supporting them through end-of-life and grief. Their unique approach thus needs to be recognized, supported by public authorities, and expanded to new geographic locations worldwide.

## Supplemental Material

sj-docx-1-pcr-10.1177_26323524251383031 – Supplemental material for Profiles of palliative day care programs in Canada and the United Kingdom: A meta-synthesis Supplemental material, sj-docx-1-pcr-10.1177_26323524251383031 for Profiles of palliative day care programs in Canada and the United Kingdom: A meta-synthesis by Gabrielle Fortin, Gabrielle Leblanc-Huard, George Kernohan and Felicity Hasson in Palliative Care and Social Practice
