# Supplementary material for: Profiles of palliative day care programs in Canada and the United Kingdom: A meta-synthesis
Source: Palliat Care Soc Pract. 2025 Oct 14;19:26323524251383031. doi: 10.1177/26323524251383031 (PMC12531439; doi:10.1177/26323524251383031)
Supplement: sj-docx-1-pcr-10.1177_26323524251383031 – Supplemental material for Profiles of palliative day care programs in Canada and the United Kingdom: A meta-synthesis [file sj-docx-1-pcr-10.1177_26323524251383031.docx]

**PROFILES OF PALLIATIVE DAY CARE PROGRAMS IN CANADA AND THE UNITED KINGDOM: A meta-synthesis**

Fortin, G., Leblanc-Huard, G., Kernohan, G. and Hasson, F.

**ENTREQ checklist**

Based on : Tong A, Flemming K, McInnes E, et al. Enhancing transparency in reporting the synthesis of qualitative research: ENTREQ. *BMC Med Res Methodol* 2012; 12: 181. 20121127. DOI: 10.1186/1471-2288-12-181.

| **No** | **Item** | **Guide and description** | **Checklist** | **Comment** |
| --- | --- | --- | --- | --- |
| **1** | Aim | State the research question the synthesis addresses. | x | The questions of the meta-synthesis are in the methodology at page 4. They are the following : 1) are there common foundations and values between PDCPs across continents? and 2) how can the evolution of UK and Canadian PDCPs help identify future avenues for the recognition and development of PDCPs? |
|  |  |  |  |  |
| **2** | Synthesis methodology | Identify the synthesis methodology or theoretical framework which underpins the synthesis, and describe the rationale for choice of methodology *(e.g. meta-ethnography, thematic synthesis, critical interpretive synthesis, grounded theory synthesis, realist synthesis, meta-aggregation, meta-study, framework synthesis).* | x | The synthesis is based on an analytic expansion approach that was realized through a thematic analysis of the data. |
|  |  |  |  |  |
| **3** | Approach to searching | Indicate whether the search was pre-planned (*comprehensive search strategies to seek all available studies)* or iterative (*to seek all available concepts until they theoretical saturation is achieved)*. |  | Since this meta-synthesis focused on secondary analysis of two studies, no literature search was conducted to produce a systematic review. This item does not apply to our paper. |
|  |  |  |  |  |
| **4** | Inclusion criteria | Specify the inclusion/exclusion criteria *(e.g. in terms of population, language, year limits, type of publication, study type).* |  | Since this meta-synthesis focused on secondary analysis of two studies, no literature search was conducted to produce a systematic review. This item does not apply to our paper. |
|  |  |  |  |  |
| **5** | Data sources | Describe the information sources used (e.g. *electronic databases (MEDLINE, EMBASE, CINAHL, psycINFO, Econlit), grey literature databases (digital thesis, policy reports), relevant organisational websites, experts, information specialists, generic web searches (Google Scholar) hand searching, reference lists)* and when the searches conducted; provide the rationale for using the data sources. | x | The source of the data was the original data of the two studies realized by the UK and Canadian research teams. The rationale choosing a secondary analysis of two studies is clearly stated on page 4. |
|  |  |  |  |  |
| **6** | Electronic Search strategy | Describe the literature search *(e.g. provide electronic search strategies with population terms, clinical or health topic terms, experiential or social phenomena related terms, filters for qualitative research, and search limits)*. |  | Since this meta-synthesis focused on secondary analyses of two studies, no literature search was conducted to produce a systematic review. This item does not apply to our paper. |
|  |  |  |  |  |
| **7** | Study screening methods | Describe the process of study screening and sifting *(e.g. title, abstract and full text review, number of independent reviewers who screened studies).* |  | Since this meta-synthesis focused on secondary analysis of two studies, no literature search was conducted to produce a systematic review. This item does not apply to our paper.. |
|  |  |  |  |  |
| **8** | Study characteristics | Present the characteristics of the included studies *(e.g. year of publication, country, population, number of participants, data collection, methodology, analysis, research questions).* | x | This information about the two studies included in the meta-synthesis is presented on pages 4. |
|  |  |  |  |  |
| **9** | Study selection results | Identify the number of studies screened and provide reasons for study exclusion *(e,g, for comprehensive searching, provide numbers of studies screened and reasons for exclusion indicated in a figure/flowchart; for iterative searching describe reasons for study exclusion and inclusion based on modifications t the research question and/or contribution to theory development).* |  | Since this meta-synthesis focused on secondary analysis of two studies, no literature search was conducted to produce a systematic review. This item does not apply to our paper. |
|  |  |  |  |  |
| **10** | Rationale for appraisal | Describe the rationale and approach used to appraise the included studies or selected findings *(e.g. assessment of conduct (validity and robustness), assessment of reporting (transparency), assessment of content and utility of the findings).* |  | Since this meta-synthesis focused on secondary analysis of two studies, no literature search was conducted to produce a systematic review. This item does not apply to our paper. |
|  |  |  |  |  |
| **11** | Appraisal items | [State the tools, frameworks and criteria used to appraise the studies or selected findings (e.g. Existing tools: CASP, QARI, COREQ, Mays and Pope [25]; reviewer developed tools; describe the domains assessed: research team, study design, data analysis and interpretations, reporting).](https://pmc.ncbi.nlm.nih.gov/articles/PMC3552766/table/T1/#B25) |  | Since this meta-synthesis focused on secondary analysis of two studies, no literature search was conducted to produce a systematic review. This item does not apply to our paper. |
|  |  |  |  |  |
| **12** | Appraisal process | Indicate whether the appraisal was conducted independently by more than one reviewer and if consensus was required. |  | Since this meta-synthesis focused on secondary analysis of two studies, no literature search was conducted to produce a systematic review. This item does not apply to our paper. |
|  |  |  |  |  |
| **13** | Appraisal results | Present results of the quality assessment and indicate which articles, if any, were weighted/excluded based on the assessment and give the rationale. |  | Since this meta-synthesis focused on secondary analysis of two studies, no literature search was conducted to produce a systematic review. This item does not apply to our paper. |
|  |  |  |  |  |
| **14** | Data extraction | Indicate which sections of the primary studies were analysed and how were the data extracted from the primary studies? *(e.g. all text under the headings “results /conclusions” were extracted electronically and entered into a computer software).* |  | Since this meta-synthesis focused on secondary analysis of two studies, no literature search was conducted to produce a systematic review. This item does not apply to our paper. |
|  |  |  |  |  |
| **15** | Software | State the computer software used, if any. | x | No computer software was used. |
|  |  |  |  |  |
| **16** | Number of reviewers | Identify who was involved in coding and analysis. | x | It is mentionned on page 6 that the canadian researchers did the coding and analysis. The UK researchers then reviewed the analysis. |
|  |  |  |  |  |
| **17** | Coding | Describe the process for coding of data *(e.g. line by line coding to search for concepts).* | x | The coding of the data process is described on pages 4 and 5. |
|  |  |  |  |  |
| **18** | Study comparison | Describe how were comparisons made within and across studies *(e.g. subsequent studies were coded into pre-existing concepts, and new concepts were created when deemed necessary).* | x | The processus of comparison is described on pages 4 and 5. |
|  |  |  |  |  |
| **19** | Derivation of themes | Explain whether the process of deriving the themes or constructs was inductive or deductive. | x | The process of deriving themes followed a mixed model and was both deductive and inductive, as described on pages 4 and 5. |
|  |  |  |  |  |
| **20** | Quotations | Provide quotations from the primary studies to illustrate themes/constructs, and identify whether the quotations were participant quotations of the author’s interpretation. | x | Quotations from the primary studies are included in the result section from page 6 to 12. |
|  |  |  |  |  |
| **21** | Synthesis output | Present rich, compelling and useful results that go beyond a summary of the primary studies (e.g. *new interpretation, models of evidence, conceptual models, analytical framework, development of a new theory or construct).* | x | By using an analytic expansion approach, we presented a new interpretation of the exsiting data. |
|  |  |  |  |  |
